# Tobacco chewing and associated factors among a vulnerable youth population in Sri Lanka

**DOI:** 10.1186/s12889-022-14704-6

**Published:** 2022-11-29

**Authors:** Manori Dhanapriyanka, R. D. F. C. Kanthi, Prasanna Jayasekara, Diep Hong Ha

**Affiliations:** 1 Institute of Oral Health, Ministry of Health, Colombo, Sri Lanka; 2 Ministry of Health, Colombo, Sri Lanka; 3 School of Dentistry, Faculty of Health and Behavioural Sciences, University of Queensland, Brisbane, Australia

**Keywords:** Youth, Urban slums, Smokeless Tobacco, Chewing, Sri Lanka

## Abstract

**Background:**

Tobacco in any form kills millions of people every year**.** Tobacco addiction among youth shows an increasing trend while smokeless type is becoming more common. This study aimed to describe the lifestyle of chewing smokeless tobacco among a group of high-risk youth population in Sri Lanka.

**Methods:**

A descriptive cross-sectional study was conducted among a sample of 1431 youths aged between 15 to 24 years residing in urban slums in Colombo Sri Lanka, using a cluster sampling technique combined with probability proportionate to size technique. Data were collected using an interviewer-administered questionnaire. Chewing smokeless tobacco was assessed using betel quid chewing and commercially prepared tobacco and areca nut packet chewing. Current chewer was defined as who had the practice of chewing during past 30 days.

**Results:**

The mean age of the study sample was 17.53 (95% CI: 17.40–17.65). Of the 1431 respondents, 57% were males and 43% were females. The prevalence of current smokeless tobacco chewers was 44.9% and among them 90.8% were males and 9.8% were females. Around 31.3% did not have smokeless tobacco chewing practice (Male-5.9%, Female-64.9%). Among the current smokeless tobacco chewers 21.5% chew both types of smokeless tobacco products and all of them were males. Male gender (OR 17.9; 11.4 -27.9) and ever smoking lifestyle (OR 4.4; 2.9–6.6) were significant determinants of current smokeless tobacco chewing lifestyle.

**Conclusion and recommendations:**

The study shows a high prevalence of smokeless tobacco use by youth aged between 15 to 24 years who were residing in urban slum areas in the district of Colombo, in Sri Lanka, highlighting this target group for early intervention to reduce the uptake and promote the quitting of this practice.

**Supplementary Information:**

The online version contains supplementary material available at 10.1186/s12889-022-14704-6.

## Introduction

Tobacco in any form kills millions of people. According to the World Health Organization (WHO), it kills more than 8 million of people every year. Tobacco use can be either smoking or smokeless type. Smokeless tobacco (SLT) is becoming more widespread globally due to various reasons [1, 2]. Those reasons range from simple beliefs among people as a safe alternative to smoking to gaps in SLT regulatory process [1, 3]. Many studies have identified SLT as a major risk factor for oral cancer and oral potentially malignant disorders [1, 2, 4].

Globally 273.9 million people used some form of SLT in 2019 and more than 80% of the SLT users were from South Asia region. Global prevalence of chewing SLT in 2019 was 4.72% and it showed an increasing trend. Sri Lanka ranked among the top 10 countries with high prevalence of chewing SLT, which was more common among males (13.57%) than females (5.15%) in 2019 [1]. Latest WHO non communicable disease risk factor survey conducted in Sri Lanka has revealed that there were 15.8% current SLT users and 11.7% were daily users [5].

A range of various SLT products are available globally in different names in different countries [6, 7]. The method of preparation and pattern of consumption varied markedly between countries [8]. The most common type of chewing tobacco in Sri Lanka is the betel quid and there are various other commercially prepared chewing products containing tobacco and areca nut such as pan parag/ pan masala, mawa and babul beeda [9].

Smokeless tobacco products are becoming common among youth groups even though there are limited research on that [1, 6, 10]. Few countries showed a higher prevalence in SLT use than smoking among young age groups [1]. Majority of tobacco users initiate this lifestyle in their younger age and continue this lifestyle to adult hood. These unhealthy lifestyles increase school dropouts, there by affecting the academic career. It also promotes violent and illegal behaviours of the users [11]. Moreover, use of SLT increase the risk of oral potentially malignant disorders, oral cancer, other tobacco related diseases. These Preventable risky lifestyles cause a reduction in billions of dollars to the society affecting the annual economic growth of these countries [12, 13].

Risk lifestyles such as smoking tobacco and chewing tobacco are more common among vulnerable populations especially those live in urban slums. The slums are housing units built mostly for long term use and are often single-room dwellings, compactly arranged in back-to-back rooms. Slums do not accommodate all the urban poor, nor are all slum dwellers always poor [14]. The people living in urban slums are commonly neglected and underprivileged without basic facilities and services and are having several lifestyle alterations such as changes in the diet, decrease in physical activity, increase in smoking, tobacco chewing and alcohol consumption and exposure to severe stresses [15]. Poor housing and neighborhood environment, lack of health knowledge, and poor physical and psychosocial health are some of the factors identified to influence their lifestyle alterations [16]. A study done among youth living in urban slums in Bangladesh has revealed that about 42% of young adults aged between 15–24 years were tobacco smokers and this prevalence was much higher than the youth living in other areas [16].

Chewing SLT lifestyle and its associated factors have not been researched adequately among youth as smoking [17]. There are no studies conducted among youth, residing in urban slum areas in Sri Lanka related to the chewing tobacco lifestyle. This study aimed to describe the lifestyle of SLT chewing among youth (15–24 years old) residing in the urban slum areas in the Colombo district in Sri Lanka.

## Methods

The study was a descriptive cross-sectional study, conducted in the urban slums in two divisional secretariat areas (Colombo and Thimbirigasyaya) in the Colombo district, from February 2016 to August 2016. Sample size was calculated using the prevalence of tobacco usage among youth in Sri Lanka (taken as 23%) [18] with 5% precision and 95% confidence level (*n* = Z^2^p (100-p)/d^2^) [19]. Since the study was conducted using the cluster sampling technique, to overcome the cluster effect a correction for cluster effect was carried out to increase the precision of the study (The final sample size was *N* = design effect X n, design effect = 1 + (b—1) rho, b = cluster size) [20, 21]. A Sample of 1435 youth aged between 15 to 24 years, were selected using the two-stage cluster sampling technique combined with probability proportionate to size technique. A single slum (Watta/Ward) was considered as one cluster. At the first stage grama-niladari divisions were selected and then relevant number of clusters were selected within the grama-niladari divisions. House to house community survey was conducted to select the study participants within the selected clusters. A pre-tested, validated interviewer-administered questionnaire was used to gather the relevant information. The questionnaire was developed after extensive literature review and referring to an already used questionnaire in Sri Lanka in a different setting [22] and it was adopted and modifies to study setting using an expert panel. The face, content and consensual validities were assessed.

SLT chewing was assessed using betel quid chewing and commercially prepared tobacco and areca nut packet (CPTAP) chewing. A current chewer was defined as a participant who had the chewing lifestyle during past 30 days before the survey, and it included both daily and non-daily chewers. A daily chewer was defined as a participant who had the chewing lifestyle daily and a never chewer was a participant who did not have the SLT chewing lifestyle. Those who had the chewing lifestyle but not within past 30 days were defined as non-current chewers. The definitions for the current chewer were the standard definition used in Global School Base Students Health Survey (GSHS) and Global Youth Tobacco Survey (GYTS) [23, 24].

Data collection was carried out after obtaining the written informed consent and statistical analysis was done using the SPSS version 21. The results were reported as percentages, odds ratio and 95% confidence interval. Bivariate logistic regression (using enter method) was used to identify independent factors associated with the current SLT chewing lifestyle. *P* value < 0.05 was considered as statistically significant. Ethical approval was taken from the faculty of Medicine, University of Colombo, Sri Lanka.

## Results

### Sociodemographic profile

A total of 1431 youth was included in the final sample in the study out of which 815 (57%) were males and 616 (43%) were females. The mean age of the study sample was 17.53 (95% CI: 17.40–17.65). Table 1 shows the sociodemographic profile of the study participants.

**Table 1:** Socio demographic profile of the study participants

Socio demographic characteristic		Number	%
Age *N* = 1431	15–19 years	939	65.6
	20–24 years	492	34.3
Sex *N* = 1431	Male	815	57.0
	Female	616	43.0
Ethnicity *N* = 1431	Sinhala	972	67.9
	Muslim	244	17.1
	Tamil	215	15.0
Engaged in educational activities *N* = 1431	Schooling or engaged in vocational training activities	451	31.5
	Not engaged in any educational activity	980	68.5
Marital status *N* = 1003	Unmarried	844	88.9
	Married	159	11.1
Employment Status *N* = 1003	Employed	466	32.6
	Unemployed	537	37.5

### Initiation of SLT

The mean age at initiation for, current SLT chewers was 14.96 years (SD ± 2.3 years) whereas mean ages at initiation for, current betel quid chewers were15.91 years, (SD ± 2.5 years) and current CPTAP chewers were15.69 years, (SD ± 2.4 years). The age of initiation of current SLT chewing lifestyle ranged from 10 to 21 years.

Majority of current SLT chewers (57.6%, 95% CI-53.8%-61.4%) mentioned that the reason for their first use of SLT was influence of their friends. Moreover 22.7% (95% CI-19.7%-26.1%) cited that the reason for their first use was availability of SLT readily at home due to the usage of parents or elder siblings. Another 8.1% (95% CI-6.2%-10.5%) mentioned that they just started without any special reasons and 11.5% (95% CI- 9.3%-14.5%) mentioned that they initiate SLT chewing to experience the joy.

### Prevalence of SLT chewing

**Fig. 1:**
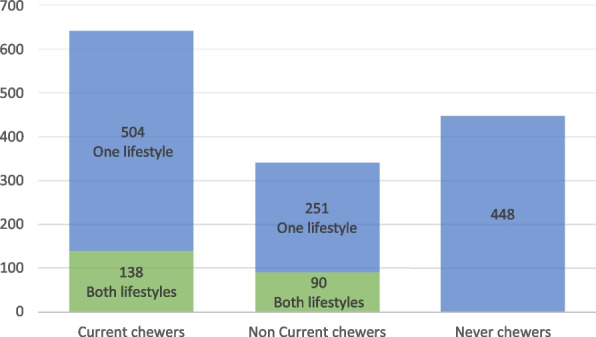
Description of the study participants according to their current SLT chewing lifestyle

The prevalence of current SLT chewers was 44.9% (*n* = 642, 95% CI- 42.3%-47.5%) and among them90.2% (*n* = 579, 95% CI- 87.6%-92.3%) were males. Around 21.5% (*n* = 138, 95% CI-18.5%—24.8%) of current SLT chewing youth had both types of chewing SLT practices and all of them were males. Further 78.5% (*n* = 504, 95%CI-75.2%-81.5%) of current SLT chewing youth had only one type of chewing SLT practice (males-87.5% and 12.5% females). Around 31.3% (*n* = 448, 95% CI – 29%-33.8%) did not have SLT chewing practices (males-10.7% and females 89.3%). Figure 1 shows description of the study participants according to their current SLT chewing lifestyle. Current SLT chewing lifestyle was significantly higher among males than females (males-90.2%, 95% CI-87.6%-92.3% and females 9.8%, 95% CI-7.7%-12.4%, *P* < 0.05). Table 2 shows the profile current SLT chewing lifestyle among study participants.

**Table 2:** The profile of current SLT chewing among study participants

Variable	No of participants with current tobacco chewing lifestyle N (%) 95% CI		No of participants with current betel chewing lifestyle N (%) 95% CI		No of participants with current CPTAP chewing lifestyle N (%) 95% CI	
**Overall Prevalence**	642 (44.9%) 42.3%-47.5%		309 (21.6%) 19.5%-23.8%		471 (32.9%) 30.5%-35.4%	
**Sex (*n* = 1431)**						
Male	579 (90.2%) 87.6%-92.3%		246 (79.6%) 74.8%-83.7%		471 (100%) 99.2%-100%	
Female	63 (9.8%) 7.7%-12.4%		63 (20.4%) 16.3%-25.2%		0 0	
	Male	Female	Male	Female	Male	Female
**Age Group (*n* = 1431)**						
20–24 years	220(34.3%) 30.7%-38.0%	8 (1.2%) 0.6%-2.4%	82 (26.5%) 21.9%-31.7%	8 (2.6%) 1.3%-5.0%	195 (41.4%) 37.0%-45.9%	0
15 to 19 years	359 (55.9%) 52.1%-59.7%	55(8.6%) 6.6%-11.0%	164 (53.1%) 47.5%-58.6%	55 (17.8%) 13.9%-22.5%	276 (58.6%) 54.1%-63.0%	0
**Marital Status (*n* = 1003)**						
Married	115 (17.9%) 15.1%-21.1%	2 (0.3%) 0.1%-1.1%	59 (19.1%) 15.1%-23.8%	2 (0.6%) 0.2%-2.3%	97 (20.6%) 17.2%-24.5%	0
Unmarried	413 (64.3%) 60.5%-67.9%	30 (4.7%) 3.3%-6.6%	154 (49.8%) 44.3%-55.4%	30 (9.7%) 6.3%-13.5%	352 (74.7%) 70.6%-78.4%	0
**Educational Status (*n* = 1431)**						
School leavers	505 (78.7%) 75.3%-81.7%	32(5.0%) 3.6%-7.0%	212 (68.6%) 63.2%-73.5%	32 (10.4%) 7.4%-14.3%	426 (90.4%) 87.5%-92.8%	0
Currently engage in education	74 (11.5%) 9.3%-14.2%	31 (4.8%) 3.4%-6.8%	34 (11.0%) 8.0%-15.0%	31 (10.0%) 7.2%-13.9%	45 (9.6%) 7.2%-12.5%	0
**Employment status(*n* = 1003)**						
Employed	331 (51.6%) 47.7%-55.4%	18(2.8%) 1.8%-4.4%	143 (46.3%) 40.8%-51.8%	18 (5.8%) 3.7%-9.0%	280 (59.4%) 55.0%-63.8%	0
Unemployed	188 (29.3%) 25.9%-32.9%	14(2.2%) 1.3%-3.6%	70 (22.7%) 18.3%-27.6%	14 (4.5%) 2.7%-7.5%	160 (34.0%) 29.8%-38.4%	0
**Ethnicity (*n* = 1431)**						
Sinhalese	368 (57.3%) 55.3%-61.1%	37 (5.8%) 4.2%-7.8%	156 (50.5%) 44.9%-56.0%	37 (12.0%) 8.8%-16.1%	305 (64.8%) 60.3%-68.9%	0
Tamils	51(7.9%) 6.1%-10.3%	13(2.0%) 1.2%-3.4%	27 (8.7%) 6.1%-12.4%	13 (4.2%) 2.5%-7.1%	37 (7.9%) 5.8%-10.6%	0
Muslims	160 (24.9%) 21.7%-28.4%	13 (2.0%) 1.2%-3.4%	63 (20.4%) 16.3%-25.2%	13 (4.2%) 2.5%-7.1%	129 (27.4%) 23.6%-31.6%	0
**Smoking Lifestyle (*n* = 1431)**						
Ever smoker	371 (57.8%) 53.9%-61.6%	17 (2.6%) 1.7%-4.2%	160 (51.8%) 46.2%-51.3%	17 (2.0%) 1.3%-3.2%	306 (65%) 60.6%-69.1%	0
Never smoker	208 (32.4%) 28.9%-36.1%	46 (7.2%) 5.4%-9.4%	86 (27.8%) 23.1%-33.1%	46 (14.9%) 11.3%-19.3%	165 (35.0%) 30.9%-39.4%	0

The betel quid chewing lifestyle was significantly higher among males. There were 79.6% (95% CI- 74.8%-83.7%) current male betel chewers compared to 20.4% (95% CI- 16.3%-25.2%) current female betel chewers (*P* < 0.05). Among the current users, 48% (95%CI- 42.43% to 53.57%) were daily chewers and the most common type of betel quid was the quid with betel leaves, tobacco, areca nut, and lime (63.6%, 95% CI 95%- 60.44% to 66.76%).

Tobacco and areca nut packet chewing (CPTAP) lifestyle was not found among current female tobacco chewers and all current CPTAP chewers were males (*p* < 0.05). Among the current CPTAP chewers, 57% (95% CI-52.53% to 61.47%) were daily chewers and the most common type used by the chewers was Mawa (71.9%, 95% CI-68.49% to 75.31%).

### Factors associated with SLT chewing lifestyle

Binary logistic regression analysis revealed that the sex, age group and the smoking lifestyle were significantly associated with the current SLT chewing lifestyle (Table 3).

**Table 3:** Factors associated with SLT chewing lifestyle

Variable	No of participants with current SLT chewing lifestyle *N* = 983		No of participants with current betel chewing lifestyle *N* = 891		No of participants with current CPTAP chewing lifestyle *N* = 666	
	OR 95%CI	*P* value	OR 95%CI	*P* value	OR 95%CI	*P* value
**Sex**						
Male	17.9 (11.4–27.9)	< 0.001	2.2(1.4–3.6)	< 0.001	^a^	
Female	Reference					
**Age Group**						
15–19 years	0.5(0.3–0.8)	< 0.001	0.3(0.2–0.5)	< 0.001	0.9(0.5–1.4)	0.72
20–24 years	Reference					
**Marital Status**						
Unmarried	1.3(0.7–2.4)	0.34	0.3(0.2–0.6)	< 0.001	1.8(1.0–3.3)	0.03
Married	Reference					
**Educational Status**						
School leavers	< 0.001	0.98	11.1(1.4–37.9)	0.02	< 0.001	0.98
Currently engage in education	Reference					
**Employment status**						
Employed	1.6(0.9–2.7)	0.97	1.7(1.0–2.8)	0.02	1.1(0.6–1.9)	0.60
Unemployed	Reference					
**Ethnicity**						
Sinhalese	4.0(2.1–7.4)	0.04	2.0(1.2–3.2)	0.99	3.5(2.0–6.2)	0.001
Muslims	3.8(1.6–8.9)	0.02	4.1(2.0–8.3)	< 0.001	1.7(0.7–4.0)	0.87
Tamils	Reference					
**Smoking Lifestyle**						
Ever smoker	4.4(2.9–6.6)	< 0.001	1.7(1.1–2.5)	< 0.001	3.2(2.1–4.9)	< 0.001
Never smoker	Reference					

^a^Sex was not included in the model because females who chewed CPTAP was 0 and in bivariate analysis it showed that male gender is significantly associated with CPTAP chewing lifestyle

## Discussion

Smokeless tobacco chewing is an emerging lifestyle among youth, and it has many adverse outcomes. STL chewing increases the risk of getting oral potentially malignant disorder which may end up in oral cancer.

The mean age for, current SLT chewing initiation was 14.96 years in the present study which was consistent with findings of Global Youth Tobacco Survey (GYTS) conducted in Sri Lanka, which explained that most tobacco users tried their first initiation of tobacco in the age group between 13–15 years [24]. Studies from Kathmandu in Nepal and Noida, and Kerala, in India were also reported that initiation age of tobacco use (smoking and chewing) as 14.15, 12.4 and 13.2 years, respectively [25]. Another study conducted among low socioeconomic population in Bangladesh has identified that the initiation age of SLT chewing was between 15 to 24 years [26]. The results of these studies were more compatible with the studies conducted in developed and other developing countries [27, 28].

In the present study, 44.9% of the study population were reported as current SLT chewers. Around 90.2% of the current SLT chewers was males as in many other studies. The prevalence of SLT usage among people above the age of 15 years varied among countries ranging from 1.1% in Thailand males to 51.5% in Myanmar males [27]. A study conducted among several European countries has found that the prevalence of SLT chewing was ranged from 2% in Finland to 12.3% in Sweden [29]. The prevalence of current SLT chewers in the present study was significantly higher compared to the prevalence of current SLT chewers in (2.4%) GYTS [24]. The difference could probably be due to different methodologies used in these studies. GYTS was a school-based survey among 13- 15 years whereas the present study was a community survey which included the both school going youth and school leavers aged 15- 24 years. This is an important finding that chances of youth having current chewed tobacco becomes higher when the age increases. The non- communicable disease risk factor survey (STEP wise approach to NCD Surveillance, STEPS) in 2015, reported that the current smokeless tobacco prevalence was 16.6% [5]. An Indian study conducted among urban slum population aged above 15 years has found that 26.8% of the smokeless tobacco users were in the age group of 15 to 24 years [30].

The National Youth Health Survey (NYHS) 2012/2013 in Sri Lanka, reported the current betel and tobacco chewing prevalence as 6.3% [31] whereas the present study found the current betel chewing prevalence as 21.6%. The definition used for current chewers was different in two surveys. Current betel and tobacco chewing was defined as usage during the past one week in NYHS as against usage during past one month. Inclusion of different age category with more school dropouts, confined to one district and inclusion of high-risk individuals could have attributed to the differences in the prevalence. Another study conducted across several countries namely Taiwan, China, Malaysia, Indonesia, Nepal and Sri Lanka have identified that the current chewing prevalence of betel quid among Sri Lankan adults above the age of 15 years as 18% for males and 13.5% for females. Across these countries the current betel chewing prevalence among men varied from 10.7% in Taiwan to 43.6% in Nepal. Among females this prevalence varied from 1.8% in China to 46.8% in Indonesia [32].

Around 32.9% current CPTAP chewers were identified among this study sample. A study conducted among adolescents aged 15 years in a rural area in Sri Lanka has identified that there were 7% current areca nut chewers and among them 1% were chewing commercially prepared areca nut packets [33]. Majority of youth were introduced to the product by their friend, which was compatible with the literature [17].

Usually most of substance abuse is common among males and that the same trend is followed with the tobacco chewers. There was significant association between current SLT chewing, and sex in binary logistic regression analysis. Similar results were reported in many studies worldwide [9, 34, 35]. Even though, Lue et all in 2011 has reported that lifetime betel chewing prevalence is significantly higher among females in Malaysia and Indonesia than males [32].

Sri Lanka was the fifth country to sign the WHO FCTC in the South East Asian region and the first country to ratify it in the region. In 2006 National Authority on Tobacco and Alcohol (NATA) act was implemented. In September 2016 act was amended and banned selling, production and import all forms of SLT products. The present study conducted before enacting the NATA act which could be one of the major reasons to have a higher prevalence of SLT usage.

Main limitation of the study was that the sample was gathered from urban slums in the district of Colombo, Sri Lanka and the results cannot be extrapolated to the entire youth residing in urban slum areas in Sri Lanka. There may be variation among youth living in urban slum areas in other parts of the country which needs to be studied further [36].

## Conclusion and recommendations

The study showed that the tobacco chewing was significantly high among youth residing in urban slum areas in the district of Colombo, Sri Lanka which was much higher than the nationally reported values for youth. Current SLT chewing lifestyle was significantly associated with sex. Targeted interventions specially focusing to empower youth with relevant skills needed to be implemented to prevent and reduce the fresh uptake of tobacco chewing behavior.

## Supplementary Information

**Additional file 1.**

## Data Availability

Available as a supplementary file.
